# Genetic Structure in a Small Pelagic Fish Coincides with a Marine Protected Area: Seascape Genetics in Patagonian Fjords

**DOI:** 10.1371/journal.pone.0160670

**Published:** 2016-08-09

**Authors:** Cristian B. Canales-Aguirre, Sandra Ferrada-Fuentes, Ricardo Galleguillos, Cristián E. Hernández

**Affiliations:** 1 Laboratorio de Genética y Acuicultura, Departamento de Oceanografía, Facultad de CienciasNaturales y Oceanográficas, Universidad de Concepción, Concepción, Casilla 160-C, Chile; 2 Laboratorio de EcologíaEvolutiva y Filoinformática, Departamento de Zoología, Facultad de CienciasNaturales y Oceanográficas, Universidad de Concepción, Concepción, Casilla 160-C, Chile; 3 Centro i~mar, Universidad de Los Lagos, Camino a Chinquihue 6 km, Puerto Montt, Chile; University of California Santa Cruz, UNITED STATES

## Abstract

Marine environmental variables can play an important role in promoting population genetic differentiation in marine organisms. Although fjord ecosystems have attracted much attention due to the great oscillation of environmental variables that produce heterogeneous habitats, species inhabiting this kind of ecosystem have received less attention. In this study, we used *Sprattus fuegensis*, a small pelagic species that populates the inner waters of the continental shelf, channels and fjords of Chilean Patagonia and Argentina, as a model species to test whether environmental variables of fjords relate to population genetic structure. A total of 282 individuals were analyzed from Chilean Patagonia with eight microsatellite loci. Bayesian and non-Bayesian analyses were conducted to describe the genetic variability of *S*. *fuegensis* and whether it shows spatial genetic structure. Results showed two well-differentiated genetic clusters along the Chilean Patagonia distribution (i.e. inside the embayment area called TicToc, and the rest of the fjords), but no spatial isolation by distance (IBD) pattern was found with a Mantel test analysis. Temperature and nitrate were correlated to the expected heterozygosities and explained the allelic frequency variation of data in the redundancy analyses. These results suggest that the singular genetic differences found in *S*. *fuegensis* from inside TicToc Bay (East of the Corcovado Gulf) are the result of larvae retention bya combination of oceanographic mesoscale processes (i.e. the west wind drift current reaches the continental shelf exactly in this zone), and the local geographical configuration (i.e. embayment area, islands, archipelagos). We propose that these features generated an isolated area in the Patagonian fjords that promoted genetic differentiation by drift and a singular biodiversity, adding support to the existence of the largest marine protected area (MPA) of continental Chile, which is the Tic-Toc MPA.

## Introduction

Marine environmental landscape parameters play an important role in promoting population genetic differentiation in marine organisms [1]. Consequently, identifying environmental parameters that promote population genetic differentiation is a major focus of study in evolutionary biology [1]. Most research on the effects of the environmental marine landscape on the genetics of population structure has been qualitative (e.g., [2,3]). However, qualitative research may not always be completely successful in identifying the factors that are responsible for the observed genetic structure of natural populations, and most importantly, they do not evaluate those environmental factors explicitly. In fact, few studies evaluate both: genetic and marine environmental data [3]. Manel et al. [4] introduced the concept of landscape genetics, which is able to explain spatial genetic patterns through landscape features (i.e. geographic, physic and chemical variables) and spatial statistics [4,5]. To date, most studies that used this approach have been performed in terrestrial organisms, leaving marine and freshwater organisms mostly unexplored [6]. Recently, concepts such as seascape genetics or marine landscape genetics have started to appear in studies that evaluate how biotic and abiotic factors promote microevolutionary processes in marine species (i.e. fishes, mollusks, crustaceans [1,3,7]). Although different marine habitats (i.e. estuary, open sea, intertidal, pelagic, benthic) could potentially affect the genetic diversity within species, fjord habitats in particular have the potential to greatly affect population genetic diversity due to the complex scenario produced by their heterogeneous geography and environmental characteristics.

Fjords are deep, high-latitude estuaries at have been excavated or modified by glaciers [8–12]. These estuaries are productive ecosystems that connect the open sea with freshwater from land drainage and melting ice [12,13]. In addition, this ecosystem has been characterized mainly by strong fluctuations in salinity, temperature, pH, oxygen [14] and ocean circulation patterns [15] such as mesoscale eddies and fronts [16]. These environmental characteristics have been indicated as drivers of population differentiation [2,16–20]. For example, there is evidence of the effect of environmental oscillations on the marine organisms of fjords at different levels of organization: changes in composition of macrobenthic and zooplankton communities [21–23], differences in mortality and growth [24,25], abundance and search efficiency [26]. Environmental factors associated with fjords (i.e. temperature, salinity, oxygen, pH, and nutrients) have been proposed as causes of trophic and reproductive adaptation [27–29], and transport and retention of larvae [30,31]. Also, other studies have found population genetics differentiation between inner and outer fjords waters [25,32,33]. In such cases, oceanographic features can be a barrier to dispersal at different ontogenetic stages, by restricting gene flow and increasing intraspecific divergence.

The Chilean Patagonian fjords constitute one of the largest fjord regions in the world, extending from latitude 41.5°S (Reloncaví Fjord) to latitude 55.9°S (Cape Horn) and covering a total of 240,000 km^2^ [12]. The geographic landscape of this region includes channels, estuaries, archipelagos, fjords, bays, peninsulas and islands [12]. In addition, this ecosystem has been characterized mainly by strong fluctuations in salinity, temperature, pH, oxygen [14] and circulation patterns [15]. The Patagonian sprat *Sprattus fuegensis* is a small pelagic marine fish of economic importance that inhabits from latitude 41°S, specifically in inner waters and fjords in the south of Chile to latitude 40°S in Argentina, including the Falkland Islands [34–38]. It lives a maximum of 6 years [39] and it is a partial spawner [38,40–42]. Female sprats mature at an average length of 13.5 cm [38] and produce pelagiceggs and larvae [13,43,44]. Thefirst developmental stages of *S*. *fuegensis* are mainly abundant in the inner waters of Chiloé Island, channels and fjords in Chile [13,43,44], and in the Atlantic Ocean they have been reported near Santa Cruz, Argentina and southward to the Falkland Islands [34,45]. We used *S*. *fuegensis* as a model to investigate how environment can shape the genetics structure of populations because: (1) it inhabits fjords and channels which have been shown to have high environmental oscillations and in general are habitats with low levels of pollution [12,14,15]; (2) it inhabits mainly the first 50 m of the water column [34–38] where environmental variables show high oscillations (see [46–48]); (3) there are no studies that evaluate seascape genetics in a fish that lives in fjords and channels in the Southern Hemisphere; (4) its geographic distribution is not only restricted to fjords and channels but extends further north into Argentina [34–38], allowing for further comparisons of genetics structure between homogeneous and heterogeneous environment; and (5) the species is economically important in Chile and further understanding of the structure of its populations will be useful in the management and conservation of stocks [49].

Based on the characteristics of the Chilean fjord, we propose that the small pelagic fish *S*. *fuegensis* has a large population genetic differentiation promoted by local fjord conditions. Given the geography of the area, we expected to find at least two genetic clusters: one group from the north of Chilean Patagonia (i.e. inner water of Chiloé (~42°-43°S)and fjords close to Aysén (~45°S), and another group in the most distant locality of the Strait of Magellan (~53° S). To test this hypothesis, we genotyped 282 adult *S*. *fuegensis* that were collected in 10 locations using eight species-specific microsatellites. We described the genetic diversity and population structure of *S*. *fuegensis* along the Chilean fjords and we evaluated the effect of marine environmental variables (i.e. temperature, salinity, oxygen, pH, nutrients, and ocean circulation pattern) that are related to the causal mechanisms (i.e. gene flow, genetic drift) of a population structure. Based on non-Bayesian and Bayesian approaches we found a strong genetic structure in this species, which is correlated with temperature ranges and nitrate concentration, two factors that could be affecting local productivity, growth rates and therefore population dynamics. Finally, we discuss how the oceanographic landscape can promote this divergence and how our results support the existence of the Marine Protected Area (MPA) located in this area (i.e. Tic-Toc MPA).

## Materials and Methods

### Sample collection

**Fig 1:**
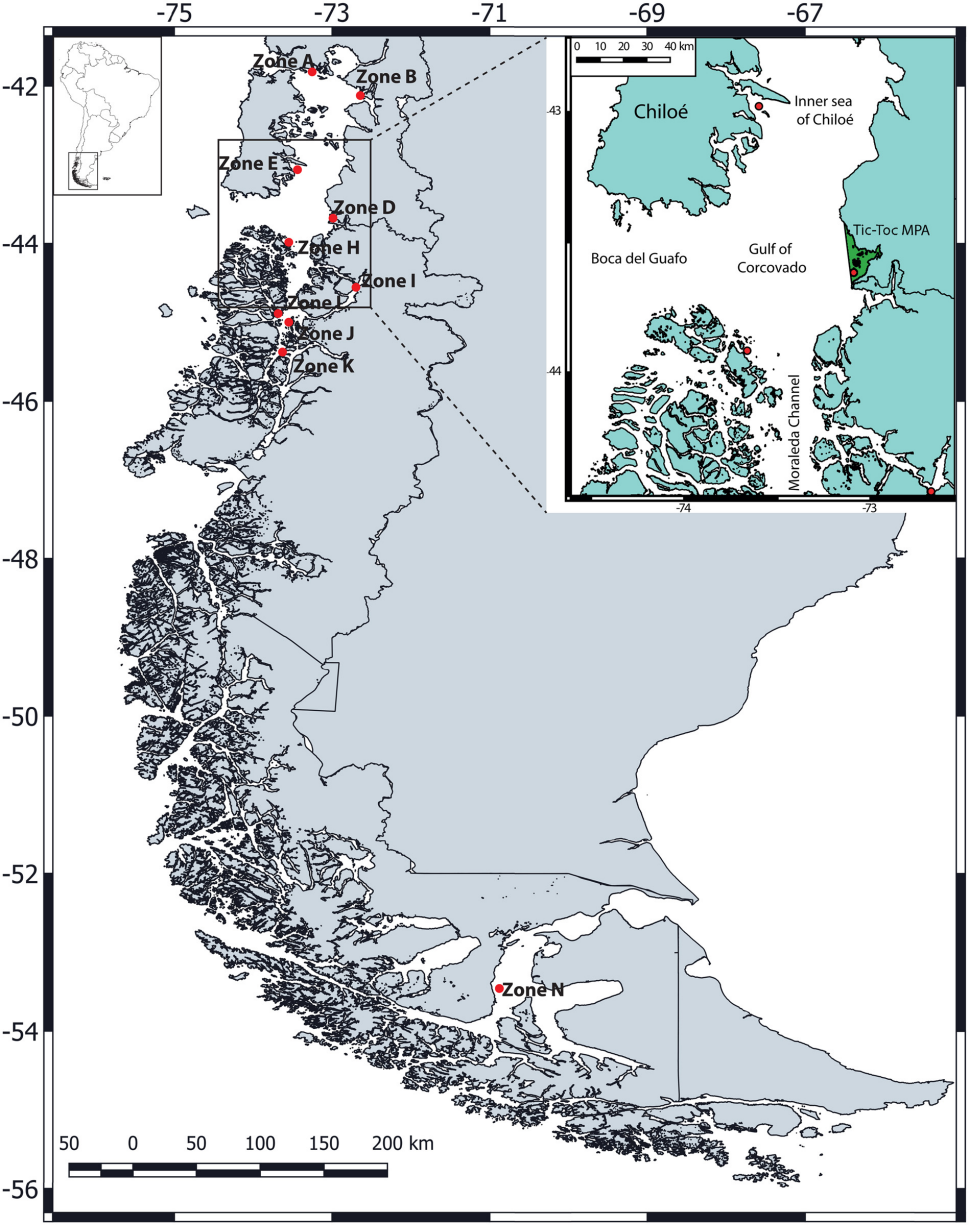
Map showing sample locations (red dots) of *Sprattus fuegensis*. Small map shows places named in the main text. The Tic-Toc MPA is shown in the green area.

A total of 282 individuals were collected from ten locations in the Chilean Patagonian fjords (Fig 1), including the inner sea of Chiloé and the particular fjords where *S*. *fuegensis* has been recorded. Locations were selected based on early studies from scientific cruises [13,50] between latitudes 41° and 46°S, except the most southern location (i.e. 53°S), which was selected based on personal communications with artisanal fishermen from Punta Arenas (i.e. in gathering information about catching sites of *S*. *fuegensis* in order to focus the sampling efforts in this area). In addition, samples collected in this southern location (i.e. 53°S) are important to the study questions given that the southern tip of South America is significantly different in temperature, phosphate and nitrate from the habitats inhabited by the northern populations (e.g. 41°S) [46–48,51–56]. Moreover, different types of vertical structures have been described for the Chilean austral channels and fjords, according to: temperature (i.e. 11°C), salinity (i.e. 7psu), dissolved oxygen-pH (i.e. 5 ml/L^-1^), phosphate-nitrate (i.e. 7 μM), and silicate (i.e. 9 μM), which support the differences among physical and chemical characteristics of the water column from Chilean Patagonia [57,58]. We sampled adults during the *S*. *fuegensis* spawning season (September and December) because this season represents the most robust period for delineating population genetic structure [59]. Sampling within this season avoids including juveniles or earlier life history stages, therefore preventing overestimating genetic differentiation by the presence of close relatives (Allendorf-Phelps effect [59,60]). In this way, we tested the null hypothesis that individuals were randomly assorted in order to spawn in different spawning areas [59,61]. Muscular tissue obtained from fillet from each individual was sampled and stored in 96% ethanol for further analyses.

### Environmental database

The currently available global marine environmental databases (e.g. BioOracle, AquaMaps, MARSPEC) have large gaps in information related to the inner sea of Chiloé and the fjords. We therefore compiled an environmental marine database based on the published literature and oceanographic research cruises. These records were obtained from the CIMAR-FIORDOS oceanographic research program conducted between 1995–2006 (http://www.shoa.cl/n_cendhoc/productos/reporte_datos.php), whose goal was to compile oceanographic and biological information from the inner sea of Chiloé, channels, estuaries and fjords from the Chilean Patagonia using the same cruise routes in different years but in similar seasons (i.e. spring—summer). The oceanographic environmental measures from the CIMAR-FIORDOS program used in this study were temperature, salinity, pH, oxygen, phosphate, and nitrate. We obtained data from the CIMAR-Fiords program for each variable, from nine different depth of the water column (i.e. 0, 2, 5, 10, 15, 20, 25, 50, and 100) in each of the 10 sampling locations. Then, we estimated the maximum, minimum, average and range in each location for the above-mentioned six marine environmental variables, in order to capture the range of conditions experienced by this species. We show the detailed information on cruises, seasons, year and references in S1 Table [46,47,51–56,62].

### Genetic database

Total genomic DNA was isolated using NucleoSpin tissue Kit (Machery-Nagel) and carried out according to the manufacturers' recommendations. The quality and quantity of DNA purification were measured in an Eppendorf biophotometer (Eppendorf AG, Hamburg, Germany) and the template DNA was diluted to 20 ng/μL for the polymerase chain reaction (PCR) amplifications. We used eight tetranucleotide microsatellites with loci described for *S*. *fuegensis* by Ferrada-Fuentes et al. [63] (i.e. Spfu_6, Spfu_9, Spfu_29, Spfu_30, Spfu_42, Spfu_44, Spfu_45, and Spfu_48). These loci were amplified following the protocol described previously by Ferrada-Fuentes et al. [63] and in the PCR procedures we included both positive and negative controls. The PCR products were run on an ABI-3130xl sequencer and sized with Naurox size standard. This was performed by locus in order to avoid a likely bias that might be generated via analyses per location. Results were analyzed using GeneMapper version 3.7 (Applied Biosystems).

### Pre-processing genetics dataset

Because large samples are expected to have more alleles than small samples, and the number of individuals per locality was not homogeneous, we conducted a rarefaction analysis to estimate how many individuals we would need in order to detect all alleles present in a population (i.e. allelic richness, A_R_) in HP-RARE [64,65]. Outputs of allelic richness obtained from rarefaction analyses indicated that the average expected number of alleles in our within-location standardized sample size (i.e. n = 12) was less than our smallest sample size obtained in the field (i.e. n = 24 to Zone_H and Zone_L), and by clusters (i.e. n = 16) was also smaller than the cluster with the lowest number of individuals sampled (i.e. n = 28 to Cluster in Zone D), therefore our number of individuals was well-suited to further analysis (Table 1).

**Table 1:** Genetic diversity parameters per sampling location and genetic cluster in microsatellite loci of *Sprattus fuegensis*.

Location	N	Lat	Long	Parameter	Spfu_6	Spfu_9	Spfu_29	Spfu_30	Spfu_42	Spfu_44	Spfu_45	Spfu_48	Mean
Zone_A	32	-41.793	-73.286	N_A_	10	17	12	14	14	21	15	12	14.4
		Inner Sea of Chiloé		H_O_	0.679	0.679	0.429	0.656	0.806	0.636	0.563	0.656	0.638
				H_E_	0.864	0.915	0.852	0.887	0.905	0.937	0.899	0.878	0.892
				F	0.214	0.258	0.497	0.26	0.109	0.321	0.374	0.253	
				H&W	0.006	0.000	0.000	0.001	0.060	0.000	0.000	0.000	
				A_R_	9	15	10	12	13	19	13	11	
Zone_B	28	-42.063	-72.860	N_A_	12	16	12	16	15	23	14	9	14.6
		Inner Sea of Chiloé		H_O_	0.636	0.679	0.148	0.852	0.75	0.857	0.75	0.571	0.655
				H_E_	0.879	0.821	0.848	0.909	0.899	0.943	0.889	0.851	0.880
				F	0.276	0.174	0.825	0.063	0.166	0.091	0.156	0.329	
				H&W	0.004	0.082	0.000	0.381	0.004	0.000	0.028	0.003	
				A_R_	11	12	11	14	13	19	12	8	
Zone_D	28	-43.860	-72.968	N_A_	10	15	12	12	12	19	9	13	12.8
		Embayment area (TicToc bay)		H_O_	0.593	0.815	0.63	0.56	0.667	0.852	0.704	0.741	0.695
				H_E_	0.636	0.767	0.826	0.834	0.861	0.855	0.785	0.862	0.803
				F	0.068	-0.062	0.238	0.329	0.225	0.004	0.104	0.141	
				H&W	0.040	0.048	0.001	0.000	0.004	0.379	0.002	0.130	
				A_R_	8	11	10	11	11	15	8	11	
Zone_E	28	-43.095	-73.675	N_A_	14	16	10	21	14	25	16	11	15.9
		Inner Sea of Chiloé		H_O_	0.75	0.667	0.481	0.692	0.786	0.654	0.643	0.643	0.665
				H_E_	0.87	0.91	0.813	0.936	0.904	0.942	0.89	0.888	0.894
				F	0.138	0.267	0.408	0.26	0.131	0.306	0.277	0.276	
				H&W	0.024	0.001	0.000	0.000	0.025	0.000	0.002	0.002	
				A_R_	12	14	9	18	13	21	13	11	
Zone_H	24	-44.091	-73.796	N_A_	12	16	8	13	15	20	12	10	13.3
		Moraleda Channel (close to Guaitecas islands)		H_O_	0.87	0.739	0.5	0.792	0.765	0.5	0.667	0.625	0.682
				H_E_	0.887	0.879	0.665	0.903	0.917	0.936	0.868	0.814	0.859
				F	0.019	0.159	0.248	0.123	0.166	0.466	0.232	0.232	
				H&W	0.557	0.030	0.127	0.071	0.002	0.000	0.003	0.010	
				A_R_	11	14	7	12	15	18	11	9	
Zone_I	30	-44.688	-72.980	N_A_	10	15	11	16	15	26	14	11	14.8
		Puyuhuapi Fjord		H_O_	0.759	0.667	0.3	0.867	0.8	0.828	0.552	0.533	0.663
				H_E_	0.862	0.899	0.861	0.922	0.913	0.955	0.897	0.865	0.897
				F	0.12	0.259	0.651	0.06	0.124	0.133	0.385	0.383	
				H&W	0.021	0.000	0.000	0.174	0.121	0.000	0.000	0.000	
				A_R_	9	13	10	14	13	22	12	10	
Zone_J	30	-45.306	-73.801	N_A_	11	23	10	13	16	24	14	11	15.3
		Meninea constriction (Fjord)		H_O_	0.767	0.621	0.4	0.767	0.815	0.69	0.679	0.69	0.679
				H_E_	0.853	0.868	0.836	0.891	0.906	0.937	0.883	0.845	0.877
				F	0.101	0.285	0.521	0.14	0.101	0.264	0.231	0.184	
				H&W	0.039	0.000	0.000	0.073	0.128	0.000	0.008	0.025	
				A_R_	10	17	9	11	14	19	12	10	
Zone_K	28	-45.767	-73.598	N_A_	10	17	7	15	17	26	12	11	14.4
		Costa Channel (Fjord)		H_O_	0.565	0.778	0.259	0.821	0.741	0.577	0.731	0.654	0.641
				H_E_	0.852	0.901	0.798	0.901	0.892	0.942	0.886	0.845	0.877
				F	0.336	0.137	0.675	0.088	0.17	0.387	0.175	0.227	
				H&W	0.001	0.008	0.000	0.085	0.002	0.000	0.005	0.012	
				A_R_	9	14	7	13	14	21	11	10	
Zone_L	24	-45.181	-73.847	N_A_	15	11	7	17	14	25	12	8	13.6
		Moraleda Channel (Fjord)		H_O_	0.75	0.435	0.25	0.591	0.957	0.75	0.87	0.75	0.669
				H_E_	0.883	0.868	0.802	0.907	0.905	0.95	0.886	0.797	0.875
				F	0.15	0.499	0.688	0.349	-0.057	0.21	0.018	0.059	
				H&W	0.040	0.000	0.000	0.000	0.427	0.000	0.264	0.468	
				A_R_	13	11	7	15	13	21	11	7	
Zone_N	30	-53.613	-70.923	N_A_	13	15	13	16	18	21	15	12	15.4
		Strait of Magellan		H_O_	0.759	0.5	0.185	0.759	0.893	0.435	0.69	0.6	0.603
				H_E_	0.885	0.893	0.877	0.9	0.927	0.933	0.908	0.872	0.899
				F	0.142	0.44	0.789	0.157	0.036	0.534	0.241	0.312	
				H&W	0.415	0.000	0.000	0.148	0.773	0.000	0.000	0.000	
				A_R_	12	13	11	13	16	19	13	10	
Cluster 1	254	-	-	N_A_	17	38	17	24	22	46	20	18	25.3
				H_O_	0.729	0.642	0.326	0.758	0.812	0.667	0.676	0.633	0.655
				H_E_	0.889	0.910	0.860	0.919	0.929	0.963	0.905	0.870	0.906
				F	0.180	0.295	0.621	0.175	0.127	0.308	0.253	0.272	
				H&W	0.000	0.000	0.000	0.000	0.000	0.000	0.000	0.000	
				A_R_	12	17	12	16	16	25	14	11	
Cluster 2	28	-	-	N_A_	10	15	12	12	12	19	9	13	12.8
				H_O_	0.593	0.815	0.630	0.560	0.667	0.852	0.704	0.741	0.695
				H_E_	0.636	0.767	0.826	0.834	0.861	0.855	0.785	0.862	0.803
				F	0.068	-0.062	0.238	0.329	0.225	0.004	0.104	0.141	
				H&W	0.011	0.011	0.001	0.000	0.001	0.192	0.000	0.041	
				A_R_	10	14	12	12	12	18	9	13	
			F_ST_(hap)	p-value	0.000	0.000	0.000	0.001	0.000	0.000	0.002	0.002	
			F_ST_	p-value	0.000	0.000	0.000	0.048	0.003	0.004	0.092	0.069	
			R_ST_	p-value	0.000	0.099	0.000	0.000	0.003	0.004	0.000	0.810	

N: sample size, Lat: Latitude, Long: Longitude, N_A_: allele number, H_O_: observed heterozygosity, H_E_: expected heterozygosity, F: fixation index, H&W: probability value associated with deviations from Hardy-Weinberg equilibrium. Bold values indicate significant differences. A_R_: allelic richness. F_ST_(hap): p-value per locus to F_ST_ index estimation from haplotype frequency, F_ST_ and R_ST_: p-values per locus to F_ST_ index estimation from distance matrix.

To evaluate the quality of the genetic database, we estimated the presence of genotyping errors such as drop-out alleles, stutter bands, and possible presence of null alleles. These analyses were conducted in the MICRO-CHECKER v2.2.3 software [66]. According to MICRO-CHECKER, several loci showed that the general excess of homozygotes is distributed across most allele size classes yielding possible deviations from Hardy-Weinberg equilibrium and presence of null alleles [66]. Taking into account that the presence of null alleles could have an impact on the estimation of population differentiation [67], and in order to avoid a decrease in power in further analyses [68], we employed model-based clustering and Bayesian assignment methods [69–71]. These methods take into account null alleles and significantly improve their accuracy in GENELAND software [70]. In addition, simulations including datasets that include the presence of null alleles have demonstrated that genetic clustering outputs do not show more gene pools than there are in reality [72] and that they improve significantly their precision in determining genetic clusters [70]. Therefore, we used the raw microsatellite dataset without any correction for null alleles to infer the number of population clusters. Then, in order to be more conservative and to achieve congruent outcomes, we tested again each locus at a time in order to remove the possible null allele effect.

Finally, in order to avoid inflating patterns of genetic structure due to sibship control (i.e. effect of sampling families [73–75]), we ruled out putative full-sibs within samples for each location. To identify full sibs we use the maximum-likelihood method implemented in COLONY v2.0.0.1 [76–78]. Full sib analysis was conducted using the ‘long length of run’ and ‘high likelihood precision’ options implemented in COLONY. The outcome from the full sibs identification analysis did not show putative full sibs in the data set, therefore we continued with further analyses without excluding any individuals from each location.

### Genetic variability

The total number of alleles (N_A_), expected (H_E_) and observed (H_O_) heterozygosity were estimated to determine the genetic variability of the samples; these parameters were calculated for each locus and locality using GENALEX v6.5 software [79]. To determine whether localities had significant deviations to the Hardy-Weinberg equilibrium and linkage-disequilibrium we conducted analyses in ARLEQUIN v3.1 [80] and GENEPOP 3.1 [81,82], respectively. Pairwise F_ST_ and R_ST_ comparisons between sampling locations were obtained from ARLEQUIN where the p-value was obtained after 10,100 permutations. In addition, two standardized measures of genetic differentiation were included in order to infer demographic processes such as genetic drift and migration on genetic population structure, as suggested by Meirmans and Hedrick [83]. Sequential Bonferroni correction [84] for multiple comparisons was applied when necessary.

### Number of genetic clusters

To infer genetic cluster number (K) in our sample set, we used two Bayesian approaches based on the clustering method which differed in that they: a) incorporated or not a null allele model, and b) useda non-spatial or spatial algorithm. We selected this approach because Bayesian models capture genetic population structure by describing the genetic variation in each population using a separate joint posterior probability distribution over loci, therefore they incorporate uncertainty into the analyses. We used STRUCTURE v.2.3.3 [85,86], which does not incorporate a null allele model, but uses a non-spatial model based on a clustering method and it is able to quantify the individual genome proportion from each inferred population. A previous run had been carried out to define what ancestry models (i.e. no admixture model and admixture model) and allele frequency models (i.e. correlated and uncorrelated allele frequency models) fit our dataset. All these previous runs were conducted with locality information prior to improving the detection of structures when these could be weak [87]. The parameters of previous simulations included five runs with 50,000 iterations following a burn-in period of 5,000 iterations for K = 1–10 as number of tested clusters. Before choosing models with which to run our dataset, we evaluated Evanno’s index ΔK [88] to identify whether different models yielded different K values, implemented in STRUCTURE HARVESTER [89]. Finally, to choose the best model to run our data we comparedthe marginal likelihood of each model which was evaluated using Bayes Factor (BF). The best two models were the no admixture and correlated frequency allele, in which sampling locations were setas an informative prior (S2 Table). These models were used in further analyses. Because the admixture model could have more biological sense, was also tested the analyses with it, but it did not yield significantly different results. The final simulations were run, testing k = 2, with 500,000 iterations of burn-in and a run length of 1,000,000 and all these were replicated ten times independently. Then, we used GENELAND v.0.3 [90], which incorporates geographic information (i.e. coordinates) in a spatial model in order to detect spatial discontinuities among populations with possible uncertainty in spatial coordinates [91] and a null allele model that improves significantly their accuracy to inferences [70]. Similarly to the methods with STRUCTURE as described above, we ran short analyses to determine what model (i.e. correlated or uncorrelated frequency models) would best fit our dataset. All runs were performed using the “null allele model” setting, given that it may have been present in our data. Previous simulations were run for testing K = 1–10, using 1,000,000 Markov chain Monte Carlo (MCMC) iterations, with a thinning interval of 10,000 and all those runs were replicated five times. The selection of the best model was evaluated using BF. The best model used was the correlated frequency model (S2 Table). The final simulations were run testing K = 2, using 10,000,000 MCMC iterations with a thinning interval of 10,000 and all those that were run were replicated ten times each. To identify the number of genetic clusters present in our data we made a graphic with density probability, per each K, per iteration. Finally, we plotted a posterior probability map of distribution in our sampling area.

### Correlations environmental and genetics

To identify patterns of population genetic variation that derive from spatially-limited gene flow (i.e. isolation by distance, IBD), we conducted a Mantel test using a transformed genetic matrix (i.e. F_ST_/(1-F_ST_) and R_ST_/(1-R_ST_)), and geographic distance (i.e. logarithms of the linear distance between locations). Pearson correlation coefficients (i.e. r) were calculated in the VEGAN package of R functions [92], and p-values were calculated on 10,000 permutations. To identify average genetic diversity parameters (i.e. N_A_, H_O_, H_E_) that show correlations with average environmental variables (i.e. temperature, salinity, pH, oxygen, phosphate and nitrate), we conducted correlation analyses in the VEGAN package of R functions.

Environmental factors that promote changes at the microevolutionary level (i.e. population genetic structure) were estimated using hierarchical Bayesian models. We conducted analyses in GESTE v2 [93], in order to evaluate whether variables from our marine environmental dataset explain patterns of population genetics structure (specific factors and dataset used in GESTE were described above). Explicitly, GESTE relates F_ST_ values with environmental factors using a generalized linear model (GLM). We ran ten pilot runs (burn-in period) to have priors of mean and variance in the distribution of alpha parameters (alpha is the vector of regression coefficients that correspond to environmental data). After these pilot runs, we ran 10,000 MCMC iterations with a thinning interval of 100 and all those runs were replicated five times each. In all, combinations of marine environmental variables were considered and evaluated using estimates of posterior probability, and the degree of uncertainty of the estimations was measured by the 95% highest probability density interval (HPDI) [93]. In order to identify whether environmental variables could explain variations in allele frequencies among locations we conducted a redundancy analysis (RDA) in the VEGAN package of R functions. Specifically, we identified the relative contribution of each environmental variable on the allelic frequency variation using a forward stepwise selection (i.e. *ordistep* function) with the Akaike information criterion in VEGAN. P-values were estimate based on 10,000 permutations. The Pearson coefficient correlation (*r*) was estimated for only the environmental variables that better explain the data variability, in order to fulfill the non-correlation for multivariate analysis [94]. Finally, we plotted these environmental variables via the *ordistep* function.

## Results

Overall, high genetic variability at all microsatellite loci were found for *S*. *fuegensis* samples, where the N_A_ per locus ranged from 7 to 26, H_E_ ranged from 0.636 to 0.955, and H_O_ ranged from 0.148 to 0.957 (Table 1). The samples from Zone_D, located inside the TicToc Bay (East of the Corcovado Gulf, Fig 1) showed the lowest mean values of N_A_ (12.8) and H_E_ (0.803) (Table 1), and the surrounding Zone_E, located in the inner sea of Chiloé (North of the Corcovado Gulf, Fig 1), the highest values (see Table 1). Pairwise F_ST_ and R_ST_ indices showed a highly significant difference in comparison between Zone_D and the remainder locations (Table 2). The standardized measure of population differentiation F'_ST_ and D_ST_ showed similar proportional magnitudes with respect to F_ST_, and R_ST_, where Zone D is the most divergent among sampling locations (S3 Table).

**Table 2:** Pairwise F_ST_ and R_ST_ indices between sampling locations.

	Zone_A	Zone_B	Zone_D	Zone_E	Zone_H	Zone_I	Zone_J	Zone_K	Zone_L	Zone_N
F_ST_										
Zone_A										
Zone_B	0.000									
Zone_D	**0.048**	**0.056**								
Zone_E	0.001	0.006	**0.051**							
Zone_H	0.004	0.002	**0.057**	0.007						
Zone_I	0.000	0.006	**0.054**	0.005	0.005					
Zone_J	0.003	0.003	**0.057**	0.010	0.009	0.003				
Zone_K	0.006	0.009	**0.052**	0.004	0.008	0.003	0.010			
Zone_L	0.000	0.000	**0.064**	0.005	0.005	0.004	0.012	0.003		
Zone_N	0.001	-0.002	**0.057**	0.004	0.006	0.001	0.004	0.009	0.003	
R_ST_										
Zone_A										
Zone_B	-0.012									
Zone_D	**0.086**	**0.063**								
Zone_E	0.009	0.061	**0.119**							
Zone_H	-0.024	0.038	**0.116**	0.009						
Zone_I	-0.029	0.031	**0.087**	-0.002	0.007					
Zone_J	0.027	**0.100**	**0.128**	-0.013	0.013	0.018				
Zone_K	-0.033	0.028	**0.084**	0.021	0.010	0.005	0.050			
Zone_L	0.039	0.079	**0.144**	0.002	0.027	0.007	0.019	0.040		
Zone_N	0.013	0.028	**0.120**	0.011	-0.047	0.002	0.023	0.007	0.021	

Bold values represent P-values less than 0.05.

Significant deviations from the Hardy-Weinberg equilibrium were found at loci for some locations of samples due to homozygote excess as indicated by MICROCHECKER outcomes (Table 1). No pairwise comparison locus seems to be in linkage disequilibrium (*P*> 0.05).

**Fig 2:**
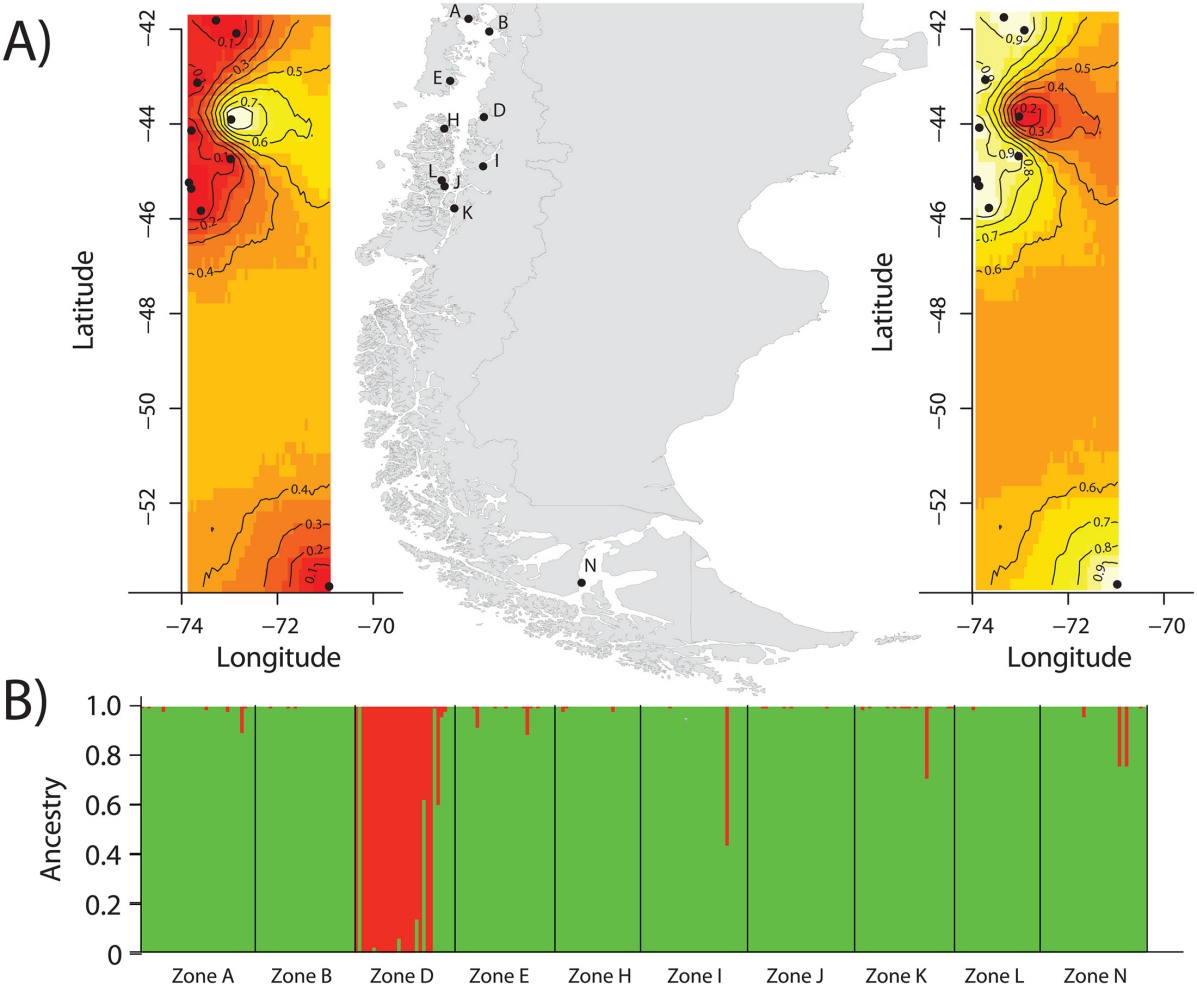
Bayesian clustering results from STRUCTURE and GENELAND. A) Plot shows the most likely number of clusters for the dataset. GENELAND analyses with posterior probability isoclines denoting the extent of genetic landscapes. Clusters indicated by GENELAND: B) Largest Cluster (LC) and C) Smallest Cluster (SC). Black dots represent localities analyzed in this study (represented by its respective letter) and regions with the greatest probability of inclusion are shown in white, whereas diminishing probabilities of inclusion are proportional to the degree of coloring.

Bayesian approaches based on the clustering method were congruent among them, despite the fact that STRUCTURE does not include a model that incorporates locus with possible null allele as in GENELAND. In addition, analyses that were run by each locus at a time showed a convergence in outcomes and found the same two clusters in 5 out of 8 loci (S4 Table). The smallest values of K that capture the major structure of the data were 2 in all of the cases (Fig 2): One genetic cluster (i.e. the major cluster, termed largest cluster, LC hereafter) includes almost all sampling localities (i.e. Zone_A, Zone_B, Zone_E, Zone_H, Zone_I, Zone_J, Zone_K, Zone_L, Zone_N); and another genetic cluster includes only Zone_D, a locality from the embayment area inside of the Tic-Toc MPA (East of the Corcovado Gulf; termed smallest cluster, SC hereafter). A similar outcome plot of membership was obtained even by using the admixture ancestry model (S1 Fig). The outcome plot from STRUCTURE revealed six individuals sampled from SC show a high genome proportion (>60%to multilocus genotype) from LC (Fig 2). Maps of posterior probabilities of population membership obtained from GENELAND to the SC and LC (Fig 2) showed the highest-probability lines (i.e. > 0.8 posterior probability), indicating the potential spatial position of genetic discontinuities between SC and LC at the mouth of Tic-Toc Bay. In addition, outcomes from GENELAND do not present any ghost populations at non-sampled areas, which means that all individuals where assigned to the number of populations inferred by the MCMC algorithm used by GENELAND.

Transformed pairwise genetic distances between locations (F_ST_/(1-F_ST_) and R_ST_/(1-R_ST_)) and the natural log of geographic distance did not reveal any association of genetic distances with geography in the Mantel tests: low, non-significant negative correlations between distance matrices (i.e. F_ST_ (r = -0.1682, P = 0.7811) and R_ST_ (r = -0.1426, P = 0.6973)) were inferred, indicating the absence of isolation by distance, even when performing a posterior analysis and excluding the divergent zone D (i.e. F_ST_ (r = -0.3466, P = 0.9494) and R_ST_ (r = -0.0845, P = 0.5808)). The coefficient of determination (R^2^) between genetic diversity indices showed values rangingfrom 0.03 to 0.48. The relationship between H_O_ and the average temperature was the highest correlation with a Pearson correlation coefficient (r) of 0.69.

The marine environmental factors that showed the highest sum of posterior probability included in the analyses were nitrate average and minimum; oxygen maximum; temperature maximum and range; and finally phosphate average, minimum and range (S5 Table). All these factors seem to be important in describing allelic frequency variation. Notwithstanding, none of the environmental variables showed a high sum of posterior probability, and the null model always explained more than 44% of the genetic structure among locations in each dataset (Table 3).

**Table 3:** Posterior probabilities of the three most probable models for the analyses including all the factors tested.

Dataset	Pr	Factor included
Average	0.54	Null
	0.07	Null, pH
	0.06	Null, Phosphate
Maximum	0.58	Null
	0.06	Null, Oxygen
	0.06	Null, Temperature
Minimum	0.53	Null
	0.08	Null, Phosphate
	0.06	Null, Salinity
Range	0.44	Null
	0.07	Null, Temperature
	0.04	Null, Salinity

Models are listed in decreasing order of posterior probabilities.

**Fig 3:**
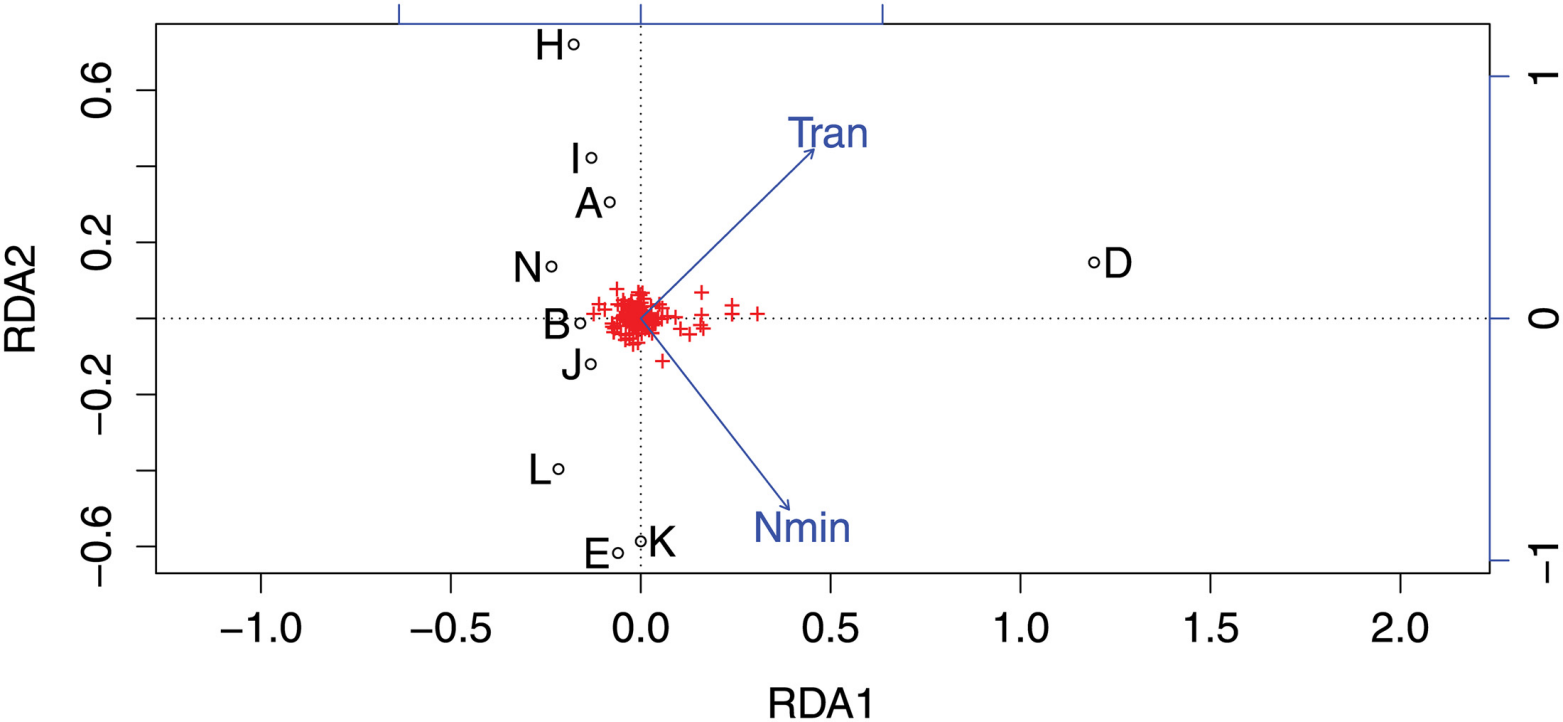
Redundancy analyses based on factors that show less Akaike value from ordistep analyses. P-value was 0.039 (p<0.05). Open circles correspond to each zone, which are represented with its respective letter. Red crosses represent the allelic variability in the dataset, blue arrows point in the direction of maximum correlation, and the length of the arrow varies according to the strength of the correlation. RDA axis corresponds to an ordination constraint which represents a linear combination of these variables.

Consequently, it means that no single environmental factor tested could explain the genetic structure observed in Bayesian analyses. The RDA that incorporated the significant environmental variables ordered by AIC (S6 Table) indicated that the minimum values of nitrate, and range values of temperature correlated withthe allelic frequency variation in our dataset (P < 0.039) (Fig 3). These explanatory variables were not correlated among them, where the variables showed a Pearson correlation coefficient of *r* = -0.113 among them (S7 Table) and determination coefficient of R = 0.012.

## Discussion

Our results show two genetic clusters in *Sprattus fuegensis* of the Patagonian fjords: one cluster restricted to Tic-Toc Bay (SC) and the other extending through the rest of the Chilean Patagonia (LC). We propose that the small cluster that is located at Tic-Toc MPA is the result of the singular oceanographic characteristics of this enclosed embayment. We find that temperature and nitrate correlate with the allelic frequency of the small cluster. We propose that larval retention and the effect of temperature and nitrate could be generating population genetic differentiation in this area. In addition, the spatial location of SC coincides with the location of the recently established marine protected area in Tic-Toc Bay, reinforcing the idea that this area has unique biogeographic characteristics.

### Genetic diversity along Chilean Patagonia

The genetic variability of *S*. *fuegensis* is remarkably similar to the variability obtainedusing microsatellite in marine, anadromous and freshwater fishes by DeWoody and Avise [95]. Compared to other marine organisms, the heterozygosity (Mean H_E_ = 0.80–0.90) in this study was similar to that of the congeneric *S*. *sprattus* (H_E_ = 0.82–0.89 [61]) and it was higher than the values of their relatives *Clupea pallasii* (H_E_ = 0.7–0.95, [96]) and *C*. *harengus* (H_E_ = 0.71–0.78, [2], and H_E_ 0.85–0.84, [97]).

The SC showed less allele number and less private alleles than LC (Table 1). Although this may be related to unequal sample size in each cluster, we discarded this possibility by conducting a rarefaction analysis, which showed no effects of sample size on allelic richness by location or by cluster. We propose that the low genetic variation showed by SC is promoted by larval retention, that generates low genetic flow and an enclosed small population size that is highly affected by genetic drift, which both change allele frequencies through time and therefore fixing alleles in this population. With the exception of private allele 306 at Spfu_29 locus from SC, all other private alleles showed low frequencies. In addition, some alleles from SC, despite being shared with the remaining locations, showed a 2- or 3-fold higher frequency than the remaining locations.

### Genetic structure: biological characteristics and environmental features

Although none of the predicted clusters were found, two well-structured clusters were found by both Bayesian analyses, providing strong support for two genetic populations of *S*. *fuegensis* along its Chilean Patagonia distribution. The F_ST_ and R_ST_ indices were consistent with the Bayesian analyses, which means that our results are not dependent on the approach used. The result found in this study is in contrast with the genetic homogeneity found in other marine species in the same geographic area (*Genypterus blacodes* [98]). However, our F_ST_ and R_ST_ outcomes are quite comparable to fixation indices at neutral loci obtained for relatives to Patagonian sprat *S*. *sprattus* [61,99], *Clupea harengus* [2,100–102] and *Clupea pallasi* [96,103,104]. For example, in Norwegian fjords, Glover et al. [61] found similar results in *Sprattus sprattus*, a closely related species of *S*. *fuegensis*. The authors found a small area that showed significant genetic differences between fjords versus the Southwest North Sea and the Southwest Celtic Sea. They further suggested that *S*. *sprattus* has a reduced connectivity between sea-going sprats and those found in Norwegian fjords. Nonetheless, they suggest that gene flow and demographic connectivity among the sprats inhabiting fjord locations is significant. In freshwater fishes distributed in Patagonia, it has been suggested that their genetic patterns are the result of barriers to gene flow and coastal refugees during glacial cycles (e.g. Percichthyidae [105], *Galaxias maculatus* [106]). In our case, given the current geographic distribution of *S*. *fuegensis*, and that it can tolerate a wide range of salinities, we cannot discard the hypothesis that historical refuges during the last glaciation might partly explain the observed pattern. Nonetheless, our data set based on microsatellite loci does not have the resolution to investigate this hypothesis, which should be evaluated using mitochondrial DNA.

The largest cluster (LC) found in our study extends from ~41° to ~53° LS (Fig 2). We hypothesize that the lack of genetic differentiation found in LC, in spite of the large geographic area covered, could be explained by the abundance and distribution of larvae, eggs and juveniles from nursery grounds or by the close proximity of spawning grounds along the Chilean geographic range. At present, specific spawning grounds of *S*. *fuegensis* have not been identified in its Chilean Patagonia distribution. Nonetheless, mature adults have been identified in the inner sea of Chiloé [37]. Moreover, the presence of juveniles has also been detected in the inner sea of Chiloé, and the fjord close to Aysén (i.e. between Puerto Aguirre and Estero Elefante) [50,107]. In numerous locations adjacent to the Strait of Magellan, Pacific and Atlantic Oceans on the Magellanic shelf, the presence of eggs has been discovered[13,42,44]. Using otolith microchemistry from juvenile *S*. *fuegensis* individuals, Galleguillos et al. [108] showed the presence of three different nursery grounds, which can be found in the inner sea of Chiloé, the fjord close to Aysén and in the Strait of Magellan. In the area of the Strait of Magellan and channels adjacent to the Atlantic Ocean, Sánchez et al. [42] identified the largest nursery ground of *S*. *fuegensis* along the Argentinean Patagonian coast with a juvenile production of 1.3x10^9^ individuals. Similarly, a probable explanation of the non-genetic differences found in *Genypterus blacodes* along inner waters, channels and fjords in Chilean Patagonia, was the close proximity of spawning grounds in the same study area [98]. Adult migration in *S*. *fuegensis* has not been recorded to date, however, indirect evidence (i.e. microchemistry of otoliths and parasites tags) has pointed out that an active dispersal of adults must exist between the inner sea of Chiloé and the fjord close to Aysén [108]. The same mechanism has been proposed in *Engraulis ringens* [109] and *Strangomera bentinki* [110], two small pelagic marine fishes distributed along the continental shelf. Overall, taking into account the broad distribution of eggs, larvae and juveniles that has been recorded for this species [13,42,44,50,107], we can suggest that migration via passive dispersal might be playing a key role in the lack of genetic structure found within LC.

The environmental characteristics can also explain the low genetic differentiation of LC. Water bodies and circulation patterns could be causing migration via passive dispersal [56]. Sievers and Silva [15] recorded the directionality of different bodies of water along the Patagonian Chilean sea (S2 Fig). They described in the superficial level (i.e. 0- ~30 m) a narrow estuarine water layer with low salinity that leads into the Boca del Guafo [15]. At the middle level (i.e. ~30- ~150 m), a depth where mainly *S*. *fuegensis* can be recorded, they described a broad Subantarctic body of water that goes into Boca del Guafo and then divides northward intot he inner sea of Chiloé and southward to the fjord and channels close to Aysén [15]. Therefore, there is a superficial circulation pattern through all the extend of LC that would be driving the connectivity among localities.

The smallest cluster (SC) has a restricted geographic distribution inside Tic-Toc Bay. The SC showed highly significant differences, giving strong support for its existence. Based on GESTE analyses, none of the tested environmental variables, physical (i.e. temperature) or chemical (salinity, pH, oxygen, phosphate and nitrate) of the datasets incorporated in this study were better than the null model (Table 3). The RDA showed similar results. However, minimum nitrate and range temperature were variables that explain the allelic frequency variation in the two clusters found in this study (Fig 3). We found contradictory outcomes in landscape genetics analysis using the GESTE and the RDA approaches. For Bayesian analysis conducted in GESTE, we did not found any variable(s) that fit better than the null model, which would indicate that environmental data do not have any effect on the genetic structure observed. However, Foll and Gaggiotti[93] indicated that when GESTE fails to identify the true model, the outcomes only are not conclusive. For multivariate analysis conducted in RDA, two variables showed a significant contribution of the genetic structure in Zone_D. The RDA analysis has been strongly supported as a powerful approach in landscape genetics as noted by Legendre and Fortin [94]. These kinds of contradictions were observed by Balkenhol et al. [111], when they comparing eleven methods commonly used to link landscape and genetics data which indicated that nonlinear methods in multivariate analysis have a better success rate (i.e. in our case RDA) than others, including GESTE. However, this does not mean that GESTE is unsuitable for landscape genetics analysis, but this analysis should be performed together with other approaches in order to choose optimal combinations of landscape genetics methods [111]. Therefore, we put more emphasis on RDA outcomes than GESTE outcomes in this study.

The SC was an unexpected outcome considering that, based on previous environmental information whereby we expected to find genetic differences between the more isolated areas. The SC is localized within the Chiloense Marine Ecoregion [112], an ecoregion that has been described as having an upwelling system where mesoscale processes such as eddies, fronts and plumes increase the retention of phytoplankton [113] and produce highly productive spring and autumn seasons [114,115]. A recent study showed that features such as eddies and fronts can enhance and concentrate the marine productivity which promote the generation of high quality patches in the plankton to be used by pelagic larvae, enhancing their survival [116]. Therefore, high phytoplankton and zooplankton aggregations and kelp forests provide feeding and refuge to diverse fish and invertebrate communities [113], and produce an overall pattern of high biodiversity [117,118]. Davila et al. [119] propos that the entire area functions as a large estuarine system. Accordingly, in the area of the Gulf of Corcovado-Boca del Guafo several submarine topographic features, groups of islands and coastal narrowing, determine a geographical configuration that energize and differentiate the enclosing water bodies [113], and that may be promoting the isolation of Tic-Toc Bay where the SC is located. Actually, the surface layer (0–~30m) does not enter Tic-Toc Bay (S2 Fig), which would decrease even more the gene flow with LC by decreasing the transport of pelagic larvae of *S*. *fuegensis* from outside the bay. Nonetheless, we found individuals from LC within SC and vice versa, and we propose that they are the result of adult migration between these areas, preferentially from LC to SC.

This SC is concordant with the marine protected area (MPA) created in 2014 by the Chilean government, which was based on the high biodiversity and unique biotic and abiotic features of the zone. The Tic-Toc MPA has a surface area of 97,929 ha surrounding the Corcovado Gulf (Fig 1) and according to Alvarez et al. [113] it has a high diversity and abundance of cetaceans, dolphins, and other marine mammals and it serves as a refuge for several taxa (i.e. phytoplankton, zooplankton, kelp forests, fish and invertebrate communities [113]). In addition, this zone is one of the few fjord areas in Chile where there is no aquaculture. In this area, so far, only species and ecosystem diversity have been considered. Our results provide the first evidence of the importance that this zone could have in regard to intraspecific genetic diversity, supporting even further its uniqueness and justifying its protection. In future similar studies it would be interesting to incorporate other marine organisms that show comparable and contrasting life history traits in order to investigate how the oceanographic features of this area could be determining their uniqueness. Our study also reminds us of the importance of incorporating genetic diversity in the analyses of future conservation areas whenever this information is available and to not underestimate the contribution to the preservation of biodiversity that a particular zone could providing.

In conclusion, our data show that the singular genetic differences found inside the Tic-Toc MPA are the result of genetic drift, probably due to larval retention throughout a combination of oceanographic mesoscale processes, geographical configuration, and the local effect of the environmental variables on genetic variation. These features have generated the isolated and restricted area that promoted genetic differentiation. Further analyses should be carried out to confirm this spatial genetic pattern, test whether this pattern is stable in the long term and also whether environmental features not explicitly tested in the present study (i.e. currents of water bodies) are able to better explain the population genetic structure of this species.

## Supporting Information

S1 Table Environmental variables used in our analyses. Ave: average, Rang: Range, Max: Maximum, Min: Minimum.
(DOCX)

S2 Table Bayes factor comparison among different models incorporated in STRUCTURE and GENELAND software. AdUn: admixture and uncorrelated model, AdCo: admixture and correlated model, NAdUn: no admixture and uncorrelated model, NAdCo: no admixture and correlated model.
(DOCX)

S3 Table The standardized measure of population differentiation F'_ST_ under the diagonal and D_ST_ above the diagonal. (DOCX)

S4 Table Cluster number by GENELAND assigned to each locus based on posterior probability density and assignment of each location to cluster found per locus. (DOCX)

S5 Table Sum of posterior probabilities of models that include a given factor. GESTE analyses included all 6 factors. Bold value indicates the two highest factor scores.
(DOCX)

S6 Table Relative contribution of each environmental variable tested using Akaike’s information criterion. Phos: Phosphate, Lat: Latitude, Long: Longitude, Nit: Nitrate, Oxy: Oxygen, Sal: Salinity, Tem: Temperature. Ave: average, Rang: Range, Max: Maximum, Min: Minimum. Bold values show significant p-values. Variable kept means environmental variables that explain variation in allele frequencies among locations.
(DOCX)

S7 Table Pearson coefficient per each environmental variable tested. Bold values show the correlation coefficient between environmental variables kept in the RDA analysis.
(DOCX)

S1 Fig Plots to estimate the best number of genetic clusters. A) Evanno et al. [88] plot for detecting the number of K groups that best fit the data. B) Plot of the mean likelihood L(K) and variance per K value from STRUCTURE. C) Plot of the number of populations simulated from the posterior distribution obtained with GENELAND.
(EPS)

S2 Fig Schematic map of horizontal water circulation in different depth layers, including sample locations. A) surface layer (0–~30m); B) intermediate layer (~30–~150m); and C) deep layer (~150 m to bottom of the sea). Image modified from Sievers and Silva [15]. Sample locations in black dots.
(EPS)

S1 Dataset Dataset of loci microsatellites used in this study. (XLSX)
