# Supplementary material for: Genetic Structure in a Small Pelagic Fish Coincides with a Marine Protected Area: Seascape Genetics in Patagonian Fjords
Source: PLoS One. 2016 Aug 9;11(8):e0160670. doi: 10.1371/journal.pone.0160670 (PMC4978504; doi:10.1371/journal.pone.0160670)
Supplement: S1 Table — Ave: average, Rang: Range, Max: Maximum, Min: Minimum. (DOCX) [file pone.0160670.s001.docx]

**S1Table. Environmental variables used in our analyses.** Ave: average, Rang: Range, Max: Maximum, Min: Minimum. Data from Zone_A to Zone_L were obtained from the Cruise CIMAR-FIORDOS 1 (Spring season; October, 18th to November, 11th 1995). Data from Zone_N was obtained from the Cruise CIMAR-FIORDOS 3 (Spring season, October, 9th to 23th 1998).

| **Variable** | **Parameter** | **Zone_A^1,2^** | **Zone_B^1,2^** | **Zone_D^1,2^** | **Zone_E^1,2^** | **Zone_H^1,2^** | **Zone_I^1,2^** | **Zone_J^1,2^** | **Zone_K^1,2^** | **Zone_L^1,2^** | **Zone_N^3^** |
| --- | --- | --- | --- | --- | --- | --- | --- | --- | --- | --- | --- |
| Temperature (ºC) | Ave | 10.675 | 10.550 | 10.587 | 9.875 | 10.063 | 9.813 | 9.688 | 9.438 | 9.750 | 7.063 |
|  | Rang | 1.5 | 1.0 | 2.8 | 0.2 | 1.5 | 2.0 | 0.5 | 0.5 | 0.5 | 0.5 |
|  | Max | 11.5 | 11.0 | 11.9 | 10.0 | 11.0 | 11.0 | 10.0 | 9.5 | 10.0 | 7.5 |
|  | Min | 10.0 | 10.0 | 9.1 | 9.8 | 9.5 | 9.0 | 9.5 | 9.0 | 9.5 | 7.0 |
| Salinity (psu) | Ave | 31.563 | 31.563 | 32.696 | 32.850 | 31.786 | 29.063 | 30.250 | 29.000 | 30.250 | 30.563 |
|  | Rang | 2.5 | 2.5 | 2.3 | 0.2 | 3.0 | 13.5 | 5.0 | 2.0 | 5.0 | 0.5 |
|  | Max | 32.5 | 32.5 | 33.6 | 33.0 | 33.0 | 33.5 | 33.0 | 30.0 | 33.0 | 31.0 |
|  | Min | 30.0 | 30.0 | 31.3 | 32.8 | 30.0 | 20.0 | 28.0 | 28.0 | 28.0 | 30.5 |
| Oxygen (ml/L) | Ave | 6.750 | 6.125 | 5.424 | 5.950 | 6.625 | 5.500 | 5.500 | 5.875 | 5.500 | 6.500 |
|  | Rang | 3.0 | 2.0 | 3.3 | 0.2 | 3.0 | 4.0 | 2.0 | 0.5 | 2.0 | 0.0 |
|  | Max | 8.0 | 7.0 | 7.1 | 6.0 | 8.0 | 7.0 | 6.0 | 6.0 | 6.0 | 6.5 |
|  | Min | 5.0 | 5.0 | 3.8 | 5.8 | 5.0 | 3.0 | 4.0 | 5.5 | 4.0 | 6.5 |
| pH | Ave | 7.863 | 7.763 | 7.83 | 7.770 | 7.863 | 7.788 | 7.688 | 7.725 | 7.700 | 7.750 |
|  | Rang | 0.3 | 0.2 | 0.2 | 0.0 | 0.4 | 0.5 | 0.2 | 0.1 | 0.2 | 0.1 |
|  | Max | 8.0 | 7.9 | 7.9 | 7.8 | 8.1 | 8.0 | 7.8 | 7.8 | 7.8 | 7.8 |
|  | Min | 7.7 | 7.7 | 7.7 | 7.8 | 7.7 | 7.5 | 7.6 | 7.7 | 7.6 | 7.7 |
| Phosphate (μM) | Ave | 1.300 | 1.400 | 2.15 | 1.550 | 0.950 | 1.200 | 1.500 | 1.600 | 1.500 | 0.650 |
|  | Rang | 1.0 | 1.0 | 0.4 | 0.4 | 1.2 | 2.4 | 0.8 | 0.0 | 0.8 | 0.4 |
|  | Max | 1.8 | 1.8 | 2.4 | 1.6 | 1.6 | 2.4 | 2.0 | 1.6 | 2.0 | 1.0 |
|  | Min | 0.8 | 0.8 | 2.0 | 1.2 | 0.4 | 0.0 | 1.2 | 1.6 | 1.2 | 0.6 |
| Nitrate (μM) | Ave | 11.000 | 13.500 | 22.5 | 16.000 | 9.000 | 10.000 | 15.000 | 16.000 | 15.000 | 4.500 |
|  | Rang | 16.0 | 12.0 | 4.0 | 0.0 | 20.0 | 24.0 | 8.0 | 0.0 | 8.0 | 4.0 |
|  | Max | 20.0 | 20.0 | 24 | 16.0 | 20.0 | 24.0 | 20.0 | 16.0 | 20.0 | 8.0 |
|  | Min | 4.0 | 8.0 | 20 | 16.0 | 0.0 | 0.0 | 12.0 | 16.0 | 12.0 | 4.0 |

References

1. Silva N, Calvete C, Sievers HA. Características oceanográficas físicas y químicas de canales australes chilenos entre Puerto Montt y Laguna San Rafael (Crucero Cimar-Fiordo 1). Cienc y Tecnol del Mar. 1997;20: 23–106.

2. Silva N, Calvete C, Sievers HA. Masas de agua y circulación general para algunos canales australes entre Puerto Montt y Laguna San Rafael, Chile (Crucero Cimar-Fiordo 1). Cienc y Tecnol del Mar. 1998;21: 17–48.

3. Valdenegro A, Silva N. Caracterización oceanográfica física y química de la zona de canales y fiordos australes de Chile entre el Estrecho de Magallanes y Cabo de Hornos (CIMAR 3 FIORDOS). Cienc y Tecnol del Mar. 2003;26: 19–60.
