# Supplementary material for: Genetic Structure in a Small Pelagic Fish Coincides with a Marine Protected Area: Seascape Genetics in Patagonian Fjords
Source: PLoS One. 2016 Aug 9;11(8):e0160670. doi: 10.1371/journal.pone.0160670 (PMC4978504; doi:10.1371/journal.pone.0160670)
Supplement: S2 Table — AdUn: admixture and uncorrelated model, AdCo: admixture and correlated model, NAdUn: no admixture and uncorrelated model, NAdCo: no admixture and correlated model. (DOCX) [file pone.0160670.s002.docx]

**S2 Table. Bayes factor comparison among different models incorporated in STRUCTURE and GENELAND software.** AdUn: admixture and uncorrelated model, AdCo: admixture and correlated model, NAdUn: no admixture and uncorrelated model, NAdCo: no admixture and correlated model.

| **Program** | **Ancestry model** | **Allele Frequency model** | **Best K** | **ln P(model \| data)** | **S.E.** | **AdUn** | **AdCo** | **NAdUn** | **NAdCo** |
| --- | --- | --- | --- | --- | --- | --- | --- | --- | --- |
| STRUCTURE | Admixture | Uncorrelated | 2 | -11266.032 | +/- 0.341 | - | 0.00E+00 | 7.07E+04 | 0.00E+00 |
| STRUCTURE | Admixture | Correlated | 2 | -11238.516 | +/- 0.272 | 8.91E+14 | - | 6.30E+13 | 0.00E+00 |
| STRUCTURE | No Admixture | Uncorrelated | 2 | -11270.29 | +/- 0.309 | 0.014 | 0.00E+00 | - | 0.00E+00 |
| STRUCTURE | No Admixture | Correlated | 2 | -11222.583 | +/- 0.239 | 7.41E+18 | 8.31E+09 | 5.23E+20 | - |
|  |  |  |  |  |  |  |  |  |  |
| GENELAND | No Admixture | Uncorrelated | 2 | -10532.236 | +/- ∞ |  |  | 0 | - |
| GENELAND | No Admixture | Correlated | 2 | -10493.699 | +/- ∞ |  |  | - | 5.45081E+16 |
