# Supplementary material for: Genetic Structure in a Small Pelagic Fish Coincides with a Marine Protected Area: Seascape Genetics in Patagonian Fjords
Source: PLoS One. 2016 Aug 9;11(8):e0160670. doi: 10.1371/journal.pone.0160670 (PMC4978504; doi:10.1371/journal.pone.0160670)
Supplement: S3 Table — (DOCX) [file pone.0160670.s003.docx]

**S3 Table. Cluster number by GENELAND assigned to each locus based on posterior probability density and assignment of each location to cluster found per locus.**

|  |  |  | Locality assigned to cluster found by GENELAND | | | | | | | | | |
| --- | --- | --- | --- | --- | --- | --- | --- | --- | --- | --- | --- | --- |
| Locus | Cluster by GENELAND |  | Zone A | Zone B | Zone D | Zone E | Zone H | Zone I | Zone J | Zone K | Zone L | Zone N |
| Spfu_6 | 2 |  | 1 | 1 | 2 | 1 | 1 | 1 | 1 | 1 | 1 | 1 |
| Spfu_9 | 2 |  | 1 | 1 | 2 | 1 | 1 | 1 | 1 | 1 | 1 | 1 |
| Spfu_29 | 3 |  | 1 | 1 | 2 | 3 | 3 | 3 | 3 | 3 | 3 | 1 |
| Spfu_30 | 2 |  | 1 | 1 | 2 | 1 | 1 | 1 | 1 | 1 | 1 | 1 |
| Spfu_42 | 2 |  | 1 | 1 | 1 | 1 | 2 | 1 | 1 | 1 | 1 | 1 |
| Spfu_44 | 3 |  | 1 | 3 | 2 | 3 | 1 | 3 | 3 | 3 | 3 | 1 |
| Spfu_45 | 2 |  | 1 | 1 | 2 | 1 | 1 | 1 | 1 | 1 | 1 | 1 |
| Spfu_48 | 1 |  | 1 | 1 | 1 | 1 | 1 | 1 | 1 | 1 | 1 | 1 |
