# Supplementary material for: Genetic Structure in a Small Pelagic Fish Coincides with a Marine Protected Area: Seascape Genetics in Patagonian Fjords
Source: PLoS One. 2016 Aug 9;11(8):e0160670. doi: 10.1371/journal.pone.0160670 (PMC4978504; doi:10.1371/journal.pone.0160670)
Supplement: S4 Table — (DOCX) [file pone.0160670.s004.docx]

**S4 Table. Sum of posterior probabilities of models that include a given factor.** GESTE analyses included all 6 factors. Bold value indicates the two highest factor scores.

|  | Sum of posterior probabilities | | | |
| --- | --- | --- | --- | --- |
| Factor | Average | Maximum | Minimum | Range |
| Temperature | 0.075 | **0.103** | 0.066 | **0.185** |
| Salinity | 0.088 | 0.078 | 0.106 | 0.134 |
| Oxygen | 0.089 | **0.115** | 0.076 | 0.147 |
| pH | 0.119 | 0.091 | 0.090 | 0.143 |
| Phosphate | **0.131** | 0.085 | **0.181** | **0.200** |
| Nitrate | **0.126** | 0.088 | **0.130** | 0.123 |
