# Supplementary material for: Genetic Structure in a Small Pelagic Fish Coincides with a Marine Protected Area: Seascape Genetics in Patagonian Fjords
Source: PLoS One. 2016 Aug 9;11(8):e0160670. doi: 10.1371/journal.pone.0160670 (PMC4978504; doi:10.1371/journal.pone.0160670)
Supplement: S5 Table — GESTE analyses included all 6 factors. Bold value indicates the two highest factor scores. (DOCX) [file pone.0160670.s005.docx]

**S5 Table. Relative contribution of each environmental variable tested using Akaike's information criterion.** Phos: Phosphate, Lat: Latitude, Long: Longitude, Nit: Nitrate, Oxy: Oxygen, Sal: Salinity, Tem: Temperature. Ave: average, Rang: Range, Max: Maximum, Min: Minimum. Bold values show significant p-values. Variable kept means environmental variables that explain variation in allele frequencies among locations.

|  | All environmental factors | | |  | Excluding Nmin | | |  | Excluding Nmin and Tran | | |
| --- | --- | --- | --- | --- | --- | --- | --- | --- | --- | --- | --- |
|  | AIC | F | Pr(>F) |  | AIC | F | Pr(>F) |  | AIC | F | Pr(>F) |
| Nave | -12.62 | 20.82 | **0.04** |  | -11.51 | 10.27 | 0.35 |  | -12.94 | 10.12 | 0.57 |
| Nmax | -11.40 | 0.93 | 0.37 |  | -11.40 | 0.94 | 0.48 |  | -12.96 | 10.30 | 0.53 |
| Nmin | -12.14 | 16.16 | **0.03** |  | Variable kept | | | | | | |
| Nran | -11.28 | 0.82 | 0.67 |  | -11.40 | 0.94 | 0.46 |  | -12.96 | 10.30 | 0.52 |
| Oave | -11.60 | 11.04 | 0.33 |  | -11.19 | 0.78 | 0.75 |  | -13.02 | 10.73 | 0.44 |
| Omax | -11.24 | 0.79 | 0.75 |  | -12.56 | 19.16 | **0.02** |  | -12.82 | 0.93 | 0.62 |
| Omin | -11.27 | 0.81 | 0.66 |  | -11.33 | 0.88 | 0.61 |  | -13.04 | 10.88 | 0.41 |
| Oran | -11.24 | 0.79 | 0.72 |  | -12.00 | 14.30 | 0.13 |  | -12.83 | 0.94 | 0.61 |
| Pave | -12.68 | 21.50 | **0.03** |  | -11.49 | 10.09 | 0.37 |  | -12.86 | 0.96 | 0.64 |
| pHave | -11.52 | 10.38 | 0.42 |  | -12.57 | 19.21 | **0.02** |  | -12.99 | 10.51 | 0.45 |
| pHmax | -11.14 | 0.70 | 0.90 |  | -12.30 | 16.83 | 0.07 |  | -12.95 | 10.19 | 0.51 |
| pHmin | -11.24 | 0.79 | 0.61 |  | -11.22 | 0.80 | 0.73 |  | -13.08 | 11.09 | 0.38 |
| pHran | -11.08 | 0.65 | 0.90 |  | -11.82 | 12.76 | 0.18 |  | -13.24 | 12.31 | 0.23 |
| Pmax | -11.67 | 11.72 | 0.27 |  | -11.43 | 0.96 | 0.45 |  | -13.02 | 10.73 | 0.42 |
| Pmin | -12.37 | 18.41 | **0.05** |  | -11.46 | 0.98 | 0.43 |  | -12.59 | 0.77 | 0.77 |
| Pran | -11.20 | 0.75 | 0.67 |  | -11.10 | 0.70 | 0.85 |  | -12.95 | 10.18 | 0.51 |
| Save | -11.87 | 13.55 | 0.18 |  | -11.72 | 11.98 | 0.26 |  | -13.13 | 11.46 | 0.34 |
| Smax | -11.26 | 0.80 | 0.60 |  | -11.38 | 0.92 | 0.52 |  | -12.65 | 0.81 | 0.75 |
| Smin | -11.34 | 0.87 | 0.35 |  | -11.30 | 0.85 | 0.62 |  | -13.27 | 12.48 | 0.22 |
| Sran | -11.14 | 0.70 | 0.75 |  | -11.20 | 0.78 | 0.74 |  | -13.18 | 11.83 | 0.29 |
| Tave | -11.20 | 0.75 | 0.60 |  | -11.02 | 0.64 | 0.93 |  | -12.63 | 0.80 | 0.79 |
| Tmax | -11.52 | 10.38 | 0.25 |  | -11.56 | 10.69 | 0.33 |  | -12.61 | 0.78 | 0.81 |
| Tmin | -10.91 | 0.50 | 1.00 |  | -10.92 | 0.56 | 0.97 |  | -12.61 | 0.78 | 0.81 |
| Tran | -12.56 | 20.26 | 0.08 |  | -13.38 | 26.74 | **0.00** |  | Variable kept | | |
