# Supplementary material for: Genetic Structure in a Small Pelagic Fish Coincides with a Marine Protected Area: Seascape Genetics in Patagonian Fjords
Source: PLoS One. 2016 Aug 9;11(8):e0160670. doi: 10.1371/journal.pone.0160670 (PMC4978504; doi:10.1371/journal.pone.0160670)
Supplement: S6 Table — Phos: Phosphate, Lat: Latitude, Long: Longitude, Nit: Nitrate, Oxy: Oxygen, Sal: Salinity, Tem: Temperature. Ave: average, Rang: Range, Max: Maximum, Min: Minimum. Bold values show significant p-values. Variable kept means environmental variables that explain variation in allele frequencies among locations. (DOCX) [file pone.0160670.s006.docx]

**S6 Table. The standardized measure of population differentiation F'_ST_ under the diagonal and D_ST_ above the diagonal.**

|  | Zone_A | Zone_B | Zone_D | Zone_E | Zone_H | Zone_I | Zone_J | Zone_K | Zone_L | Zone_N |
| --- | --- | --- | --- | --- | --- | --- | --- | --- | --- | --- |
| Zone_A |  | 0.074 | 0.371 | 0.066 | 0.142 | 0.079 | 0.112 | 0.101 | 0.086 | 0.010 |
| Zone_B | -0.044 |  | 0.418 | 0.089 | 0.075 | 0.072 | 0.047 | 0.086 | 0.025 | 0.034 |
| Zone_D | 0.313 | 0.360 |  | 0.354 | 0.435 | 0.423 | 0.412 | 0.357 | 0.485 | 0.434 |
| Zone_E | -0.066 | 0.006 | 0.325 |  | 0.108 | 0.067 | 0.105 | 0.072 | 0.051 | 0.057 |
| Zone_H | 0.026 | -0.011 | 0.354 | 0.026 |  | 0.097 | 0.099 | 0.123 | 0.087 | 0.101 |
| Zone_I | -0.029 | 0.004 | 0.388 | -0.011 | 0.036 |  | 0.030 | 0.045 | 0.035 | 0.052 |
| Zone_J | 0.014 | -0.024 | 0.358 | 0.035 | 0.022 | -0.029 |  | 0.111 | 0.102 | 0.060 |
| Zone_K | -0.028 | -0.011 | 0.306 | -0.027 | 0.025 | -0.037 | 0.032 |  | 0.046 | 0.121 |
| Zone_L | -0.032 | -0.059 | 0.404 | -0.036 | 0.004 | -0.030 | 0.038 | -0.050 |  | 0.061 |
| Zone_N | -0.145 | -0.081 | 0.371 | -0.074 | -0.008 | -0.056 | -0.035 | 0.000 | -0.052 |  |
