# Supplementary material for: Genetic Structure in a Small Pelagic Fish Coincides with a Marine Protected Area: Seascape Genetics in Patagonian Fjords
Source: PLoS One. 2016 Aug 9;11(8):e0160670. doi: 10.1371/journal.pone.0160670 (PMC4978504; doi:10.1371/journal.pone.0160670)
Supplement: S7 Table — Bold values show the correlation coefficient between environmental variables kept in the RDA analysis. (DOCX) [file pone.0160670.s007.docx]

**S7 Table. Pearson coefficient per each environmental variable tested.** Bold values show the correlation coefficient between environmental variables kept in the RDA analysis.

|  | Temperature | | | |  | Salinity | | | |  | Oxygen | | | |
| --- | --- | --- | --- | --- | --- | --- | --- | --- | --- | --- | --- | --- | --- | --- |
|  | Ave | Ran | Max | Min |  | Ave | Ran | Max | Min |  | Ave | Ran | Max | Min |
| T_Ave | 1.000 |  |  |  |  |  |  |  |  |  |  |  |  |  |
| T_Rang | 0.465 | 1.000 |  |  |  |  |  |  |  |  |  |  |  |  |
| T_max | 0.943 | 0.723 | 1.000 |  |  |  |  |  |  |  |  |  |  |  |
| T_Min | 0.914 | 0.084 | 0.749 | 1.000 |  |  |  |  |  |  |  |  |  |  |
| S_Ave | 0.363 | 0.227 | 0.372 | 0.319 |  | 1.000 |  |  |  |  |  |  |  |  |
| S_Rang | 0.163 | 0.361 | 0.290 | 0.071 |  | -0.579 | 1.000 |  |  |  |  |  |  |  |
| S_Max | 0.563 | 0.504 | 0.663 | 0.474 |  | 0.442 | 0.423 | 1.000 |  |  |  |  |  |  |
| S_Min | 0.007 | -0.230 | -0.099 | 0.077 |  | 0.778 | -0.955 | -0.135 | 1.000 |  |  |  |  |  |
| O_Ave | -0.170 | -0.124 | -0.143 | -0.088 |  | 0.267 | -0.467 | -0.342 | 0.399 |  | 1.000 |  |  |  |
| O_Rang | 0.627 | 0.821 | 0.814 | 0.387 |  | 0.013 | 0.658 | 0.690 | -0.494 |  | -0.174 | 1.000 |  |  |
| O_Max | 0.394 | 0.664 | 0.585 | 0.206 |  | 0.338 | 0.075 | 0.292 | 0.014 |  | 0.602 | 0.651 | 1.000 |  |
| O_Min | -0.525 | -0.575 | -0.626 | -0.351 |  | 0.238 | -0.806 | -0.683 | 0.658 |  | 0.682 | -0.820 | -0.099 | 1.000 |
| Ph_Ave | 0.402 | 0.688 | 0.589 | 0.190 |  | 0.527 | -0.061 | 0.306 | 0.168 |  | 0.547 | 0.524 | 0.914 | 0.002 |
| Ph_Rang | 0.327 | 0.622 | 0.529 | 0.166 |  | -0.269 | 0.768 | 0.461 | -0.689 |  | 0.041 | 0.877 | 0.661 | -0.651 |
| Ph_Max | 0.444 | 0.653 | 0.630 | 0.283 |  | 0.167 | 0.344 | 0.399 | -0.245 |  | 0.428 | 0.753 | 0.922 | -0.291 |
| Ph_Min | 0.008 | -0.242 | -0.105 | 0.081 |  | 0.705 | -0.918 | -0.292 | 0.908 |  | 0.499 | -0.567 | 0.048 | 0.778 |
| P_Ave | 0.627 | 0.314 | 0.543 | 0.482 |  | 0.264 | -0.057 | 0.291 | 0.158 |  | -0.654 | 0.191 | -0.243 | -0.433 |
| P_Rang | 0.246 | 0.416 | 0.403 | 0.183 |  | -0.272 | 0.867 | 0.556 | -0.766 |  | -0.071 | 0.733 | 0.436 | -0.631 |
| P_Max | 0.685 | 0.646 | 0.760 | 0.476 |  | -0.042 | 0.654 | 0.693 | -0.488 |  | -0.701 | 0.781 | 0.104 | -0.946 |
| P_Min | 0.210 | -0.009 | 0.086 | 0.133 |  | 0.276 | -0.511 | -0.133 | 0.516 |  | -0.418 | -0.270 | -0.418 | 0.038 |
| Ni_Ave | 0.595 | 0.249 | 0.494 | 0.474 |  | 0.305 | -0.126 | 0.281 | 0.230 |  | -0.653 | 0.122 | -0.290 | -0.379 |
| Ni_Rang | 0.315 | 0.477 | 0.481 | 0.237 |  | -0.178 | 0.689 | 0.465 | -0.601 |  | 0.215 | 0.790 | 0.701 | -0.507 |
| Ni_Max | 0.847 | 0.677 | 0.909 | 0.662 |  | 0.077 | 0.581 | 0.725 | -0.397 |  | -0.468 | 0.874 | 0.347 | -0.883 |
| Ni_Min | 0.187 | **-0.113** | 0.034 | 0.157 |  | 0.259 | -0.424 | -0.068 | 0.442 |  | -0.559 | -0.350 | -0.592 | 0.013 |

Table S7: Pearson coefficient per each environmental variable tested (continued). Bold values show the correlation coefficient between environmental variables kept in the RDA analysis.

|  | pH | | | |  | Phosphate | | | |  | Nitrate | | | |
| --- | --- | --- | --- | --- | --- | --- | --- | --- | --- | --- | --- | --- | --- | --- |
|  | Ave | Ran | Max | Min |  | Ave | Ran | Max | Min |  | Ave | Ran | Max | Min |
| T_Ave |  |  |  |  |  |  |  |  |  |  |  |  |  |  |
| T_Rang |  |  |  |  |  |  |  |  |  |  |  |  |  |  |
| T_max |  |  |  |  |  |  |  |  |  |  |  |  |  |  |
| T_Min |  |  |  |  |  |  |  |  |  |  |  |  |  |  |
| S_Ave |  |  |  |  |  |  |  |  |  |  |  |  |  |  |
| S_Rang |  |  |  |  |  |  |  |  |  |  |  |  |  |  |
| S_Max |  |  |  |  |  |  |  |  |  |  |  |  |  |  |
| S_Min |  |  |  |  |  |  |  |  |  |  |  |  |  |  |
| O_Ave |  |  |  |  |  |  |  |  |  |  |  |  |  |  |
| O_Rang |  |  |  |  |  |  |  |  |  |  |  |  |  |  |
| O_Max |  |  |  |  |  |  |  |  |  |  |  |  |  |  |
| O_Min |  |  |  |  |  |  |  |  |  |  |  |  |  |  |
| Ph_Ave | 1.000 |  |  |  |  |  |  |  |  |  |  |  |  |  |
| Ph_Rang | 0.470 | 1.000 |  |  |  |  |  |  |  |  |  |  |  |  |
| Ph_Max | 0.837 | 0.835 | 1.000 |  |  |  |  |  |  |  |  |  |  |  |
| Ph_Min | 0.276 | -0.677 | -0.159 | 1.000 |  |  |  |  |  |  |  |  |  |  |
| P_Ave | -0.080 | -0.245 | -0.256 | 0.097 |  | 1.000 |  |  |  |  |  |  |  |  |
| P_Rang | 0.274 | 0.883 | 0.650 | -0.713 |  | -0.307 | 1.000 |  |  |  |  |  |  |  |
| P_Max | 0.094 | 0.500 | 0.248 | -0.565 |  | 0.660 | 0.484 | 1.000 |  |  |  |  |  |  |
| P_Min | -0.242 | -0.639 | -0.556 | 0.402 |  | 0.816 | -0.783 | 0.166 | 1.000 |  |  |  |  |  |
| Ni_Ave | -0.117 | -0.307 | -0.293 | 0.158 |  | 0.990 | -0.371 | 0.601 | 0.845 |  | 1.000 |  |  |  |
| Ni_Rang | 0.485 | 0.955 | 0.850 | -0.575 |  | -0.397 | 0.918 | 0.352 | -0.784 |  | -0.454 | 1.000 |  |  |
| Ni_Max | 0.299 | 0.623 | 0.496 | -0.454 |  | 0.569 | 0.541 | 0.936 | 0.056 |  | 0.522 | 0.520 | 1.000 |  |
| Ni_Min | -0.371 | -0.707 | -0.669 | 0.374 |  | 0.838 | -0.718 | 0.203 | 0.953 |  | 0.874 | -0.828 | 0.049 | 1.000 |
